# Modeling the Effect of Spatial Structure on Solid Tumor Evolution and Circulating Tumor DNA Composition

**DOI:** 10.3390/cancers16050844

**Published:** 2024-02-20

**Authors:** Thomas Rachman, David Bartlett, William LaFramboise, Patrick Wagner, Russell Schwartz, Oana Carja

**Affiliations:** 1 Computational Biology Department, School of Computer Science, Carnegie Mellon University, Pittsburgh, PA 15213, USA; 2 Joint Carnegie Mellon University-University of Pittsburgh Ph.D. Program in Computational Biology, Pittsburgh, PA 15213, USA; 3 Allegheny Cancer Institute, Allegheny Health Network, Pittsburgh, PA 15224, USA

**Keywords:** tumor growth model, tumor evolution, spatial evolution, ctDNA, tumor DNA shedding

## Abstract

**Simple Summary:**

As a tumor grows, DNA fragments from cancer cells shed into the bloodstream. Known as circulating tumor DNA (ctDNA), these fragments can be used to inform on cancer diagnosis, treatment, and prognosis. However, despite the potential for these uninavasive liquid biopsies to revolutionize cancer monitoring and treatment, ctDNA can show poor genetic concordance between blood and the main tumor tissue, hampering its general clinical utility. For liquid biopsy technologies and ctDNA analyses to transform cancer care, from early screening and diagnosis through treatment and long-term follow-up, we need to better understand how to interpret the genetic diversity measured in the blood and how it can be used to describe the true composition of the tumor tissue. In this work we study the evolutionary processes that can lead to genetic discordance between a blood sample and the main tumor tissue and specifically, how tumor spatial heterogeneity shapes these genetic differences. We find that spatial heterogeneity in apoptosis and cellular shedding across different regions of a tumor can significantly bias the mutational composition of ctDNA and emphasize important directions for further theoretical and clinical investigation into the effect of the microenvironment on ctDNA origin and quantification.

**Abstract:**

Circulating tumor DNA (ctDNA) monitoring, while sufficiently advanced to reflect tumor evolution in real time and inform cancer diagnosis, treatment, and prognosis, mainly relies on DNA that originates from cell death via apoptosis or necrosis. In solid tumors, chemotherapy and immune infiltration can induce spatially variable rates of cell death, with the potential to bias and distort the clonal composition of ctDNA. Using a stochastic evolutionary model of boundary-driven growth, we study how elevated cell death on the edge of a tumor can simultaneously impact driver mutation accumulation and the representation of tumor clones and mutation detectability in ctDNA. We describe conditions in which invasive clones are over-represented in ctDNA, clonal diversity can appear elevated in the blood, and spatial bias in shedding can inflate subclonal variant allele frequencies (VAFs). Additionally, we find that tumors that are mostly quiescent can display similar biases but are far less detectable, and the extent of perceptible spatial bias strongly depends on sequence detection limits. Overall, we show that spatially structured shedding might cause liquid biopsies to provide highly biased profiles of tumor state. While this may enable more sensitive detection of expanding clones, it could also increase the risk of targeting a subclonal variant for treatment. Our results indicate that the effects and clinical consequences of spatially variable cell death on ctDNA composition present an important area for future work.

## 1. Introduction

Once far-fetched, the idea that a blood sample can precisely inform cancer diagnosis, treatment, and prognosis is quickly becoming clinical reality [1]. This is largely due to advances in the quantification of DNA fragments from cancer cells shed into the bloodstream, known as circulating tumor DNA (ctDNA), which are primarily released from the tumor via apoptosis, necrosis, and active secretion [2]. While tissue biopsies have been a critical component in cancer care, providing a snapshot of the tumor–host microenvironment, they are invasive, and repeated biopsies over time to monitor cancer progression and optimize therapies are seldom feasible. Moreover, even when accessible, a single biopsy sample may not represent an entire tumor, which usually displays significant spatial heterogeneity. ctDNA-based “liquid biopsies”, on the other hand, do not have some of these drawbacks and can act as a noninvasive cancer biomarker, allowing more frequent and comprehensive analyses of the tumor’s genetic evolution [3,4,5]. Two major applications of ctDNA already used in the clinic includd the monitoring of tumor burden before, during, and after treatment and for the detection of post-treatment relapse [6,7]. Liquid biopsies have also shown great promise in predicting relapse, progression-free survival, and overall survival across a variety of tumor types and stages [3,8,9,10].

Despite its potential to revolutionize cancer monitoring and treatment, ctDNA can also show poor concordance between blood and tissue, hampering its general clinical utility [11,12]. The main causes for this include access to only minuscule concentrations of ctDNA in a plasma sample, the limits of current sequencing technologies, the confounding effects of noncancerous mutations, and intratumor heterogeneity [13]. While improvements in assay sensitivity and specificity could help to better resolve the ground-truth composition of the observed ctDNA in a blood sample, we need different methods to better understand and correct possible inaccuracies arising from biased representations of the different tumor clones in ctDNA fragments.

Changes to ctDNA yield and representation of different mutations have been observed before and during chemotherapy, altering the detectability of resistance-causing mutations [14,15,16]. The majority of cfDNA fragments are around 100–160 base pairs long, which is consistent with the apoptosis-induced digestion of nuclear DNA into fragments within the circumference of a nucleosome [17,18,19]. Elevated apoptosis can increase the amount and clinical detectability of ctDNA in the bloodstream [20], and varying apoptosis rates between clones can in theory lead them to become disproportionately represented in the bloodstream [21]. In addition to the intrinsic differences in growth and death rates for different clones, heterogeneity in the tumor microenvironment due to immune infiltration, hypoxia, or treatment onset can also significantly impact rates of apoptosis [20,22,23,24,25,26,27,28]. These can in turn influence the evolutionary fate of a tumor by altering its local selective pressures and genetic heterogeneity [29].

While there are many models for studying tumor growth and evolution, the degree to which this underlying genetic distortion between blood and tumor tissue exists and the evolutionary mechanisms that shape it are not directly considered either in models of tumor evolution derived from ctDNA [30] or in clinical studies of ctDNA concordance [31]. Recent mathematical models have been used to study how varying the apoptosis rates of tumor cells could influence the time to detection of early-stage tumors [32] or the effect of differential shedding on the representation of different metastases in ctDNA [33] but ignore the underlying evolutionary process or study neutral, nonspatial evolution. Separately, a model by Fu et al. [34] showed how reduced chemotherapy exposure in a sanctuary site can promote acquired resistance, but this work did not specifically model the effects on ctDNA genetic distortions.

Here, we combined a stochastic model of boundary-driven tumor evolution [35,36,37,38,39] with a model of differential apoptosis and cellular shedding and studied the effects of spatially heterogeneous cellular apoptosis on ctDNA composition and its genetic distortion relative to the tumor tissue. We spatially constrained tumor evolution by assuming that differential drug penetration or immune system infiltration leads to increased cell death and DNA fragment shedding on the edge of the growing tumor. We compared the results across a variety of modeling choices, such as differences between quiescent or proliferative tumors, and tracked the distortion of clones and subclonal mutations in the ctDNA over time.

We found that as cancers grow and shed DNA into the bloodstream, the clones responsible for expansion into the edge environment are consistently overrepresented in the ctDNA, and, in some cases when progression results in highly heterogeneous tumors, homogeneous regions trapped in the tumor core are under-represented in the blood. We further found that over-representation of clones from high-shedding tumor regions can lead to differences in the number of detectable subclonal driver mutations and that the chosen sequencing detection limit can have a complex effect on the extent of the observed genetic differences. We also discuss the potential clinical relevance of distortions in ctDNA genetic variability during clinically significant events, such as the appearance of an expanding subclone or cell-turnover-driven increases in clonal diversity.

For liquid biopsy technologies and ctDNA analyses to transform cancer care, from early screening and diagnosis through treatment and long-term follow-up, we need to better understand how to interpret the genetic diversity measured in the blood and how it can be used to describe the true composition of the tumor tissue. Overall, our results showcase how spatial heterogeneity in apoptosis and cellular shedding across different regions of a tumor can significantly bias the mutational composition of ctDNA and emphasize important directions for further theoretical and clinical investigation into the effect of the microenvironment on ctDNA origin and quantification.

## 2. Methods

**The tumor growth model.** While there are many models of tumor growth, to analyze the role of a solid tumor’s spatial structure in shaping the observed variation in ctDNA, we used a model of boundary-driven growth, in which cells on the periphery of a tumor are assumed to experience higher proliferation rates over time as compared to the tumor core. This type of spatially restricted growth is usually observed in tissues with weak physical resistance, and it can significantly alter tumor evolution by blunting the strength of selection, promoting clonal interference, and increasing mutation burden from the tumor core to its edges [35,38]. Because of its simplicity and well-understood properties, it is an excellent starting point for exploring how spatial variation in apoptosis can impact ctDNA release and can bias the observed genetic differences between blood and main tissue.

In our Eden model, cells grow on a 2D regular lattice and each cell has eight neighbors (a Moore neighborhood), similar to Waclaw et al. [35], Chkhaidze et al. [37], Noble et al. [38], Lewinsohn et al. [39]. Each simulation begins with a single cell and terminates when the population either becomes extinct or reaches a size of 60,000 voxels. In the initial stage of growth, the tumor experiences an environment with death rate $d_{1}$. Once the tumor reaches a large enough size (here, a radius of 90 voxels or, equivalently, 3 billion cells), we assume the tumor is detected, and treatment can occur, which can shrink the initial tumor. After detection, we assume that due to differential chemotherapy drug penetration or differences in immune infiltration and oxygenation, spatial differences in apoptosis appear between the tumor core and the edge of the tumor. Specifically, cells in the core, or the sanctuary site (radius R≤90), continue to experience death at rate $d_{1}$, while on the tumor edge, cells have death rate $d_{2}\leq d_{1}$. For the sake of simplicity, we do not model angiogenesis or the interactions of cancer cells with other cell types.

**Figure 1:**
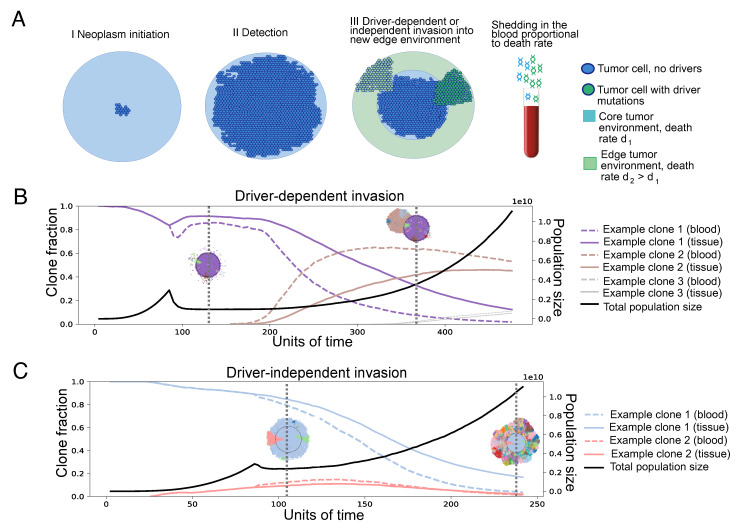
(**A**) Illustration of the model. Tumors grow to a clinically detectable size (a 2D cross-section of a 3-billion-cell tumor) and are then partially exposed to a new environment, where the cells die with rate $d_{2}$. The growth rate in the new environment determines the invasive potential of a clone. If the death rate $d_{2}$ is higher than the initial birth rate, only clones with mutations increasing the growth rate to a positive number can grow in the new environment, so invasion is driver-dependent. Otherwise, it is driver-independent. Tumor growth can be proliferative or quiescent. In the former, cells divide when they have an empty neighbor on the lattice and die at a rate independent of their neighbors. In the latter, cells also divide when they have an empty neighbor on the lattice; however, cell death also requires empty neighbors. The shedding rate of DNA into the blood is assumed to be proportionate to the death rate. (**B**) Example trajectories, driver-dependent invasion. Trajectories of clone fractions and total population size for driver-dependent invasion, with 2D visualizations of the tumor at selected timepoints. Each color corresponds to a unique clone, also shown in the trajectory plot. (**C**) Example trajectories, driver-independent invasion. Trajectories of clone fractions and total population size for driver-independent invasion, with 2D visualizations of the tumor at selected timepoints. For both cases, $\mu =0.001,s=0.1,d_{1}=0.1,b=0.7$. For driver-dependent invasion, $d_{2}=0.9$. For driver-independent invasion, $d_{2}=0.69$.

This spatial difference in death rates effectively creates a selective barrier for tumor expansion. We consider two parameter regimes: $d_{1}<b<d_{2}$ and $d_{1}<d_{2}<b$, which we call “driver-dependent” and “driver-independent” invasion, respectively (Figure 1). In the driver-dependent regime, only lineages that have acquired sufficient driver mutations can expand past the core radius *R*, while, with driver-independent invasion, all lineages continue to grow in the presence of the new edge environment. At each time step, a random cell is chosen uniformly from the population, which attempts division with a probability equal to its birth rate $b*{\left(1+s\right)}^{n}$, where *b* is the baseline birth rate in the population, *s* is the selective advantage of driver mutations, and *n* is the chosen cell’s driver mutation count. If the cell attempts division, it places a daughter cell in a randomly chosen empty site in its Moore neighborhood. If the cell is completely surrounded, it cannot divide. Upon division, we assume that the daughter cell acquires a Poisson-distributed number of additional driver mutations with rate μ. We assume each mutation appears only once (infinite site assumption). After attempting division, the chosen cell is removed from the population with probability equal to its death rate $d_{i}$, where i∈1,2 indicates which region of the tumor the cell inhabits.

We also analyzed a version of the main model where cells do not die if they are fully surrounded, so that the tumor core remains in a quiescent state and where selection acts by reducing the apoptosis rate rather than increasing birth rate, so that d←d∗(1−s).

**Parameter Choices.** To significantly reduce simulation time and memory, we assumed a Poisson distributed driver mutation rate of μ=0.001, roughly 100 times the estimated empirical rate, which we denote by $\mu_{real}=1\times 10^{-5}$, as in Bozic et al. [36]. We also simulated the tumors in 2D, so that the spatial heterogeneity reflects that of a cross-section of a much larger 3D tumor, a rationale used in Noble et al. [38] for similar 2D spatial models. Each 2D voxel then represents $\frac{\mu }{\mu_{real}}$ identical cells. For a simulation with *m* voxels, we roughly approximate the 3D tumor size, *N*, to be that of a sphere, with a cross-section equal in area to the number of 2D cells, such that $N=\frac{4}{3}\pi {\left(\frac{\mu }{\mu_{real}}\frac{m}{\pi }\right)}^{\frac{3}{2}}$. We further chose a sanctuary site radius, *R*, ranging from 20 to 60 voxels. Assuming 20 µm diameter tumor cells and 100 cells per 2D voxel, this *R* would correspond to an equivalent tumor with a radius of 0.4 to 1.2 cm and approximately 1000 to 20,000 cells, representing a cross-section of a 3D tumor of roughly 30 million–1 billion cells [40]. We simulated tumors until they expanded well beyond the core sanctuary site and stopped the simulations when tumors reached a size of 60,000 voxels, corresponding to a tumor size of approximately 10 billion cells or a radius of 2.5 cm. Without loss of generality, throughout what follows, we also assumed a constant selective benefit for driver mutations, s=0.1. See the main parameters used in the model in Table 1.

**Modeling clone fractions and variant allele frequencies (VAFs) in ctDNA.** To compute the clone frequencies in ctDNA, let $N_{ij}$ be the number of cells of clone *i* from region *j*, with corresponding death rate $d_{j}$. We assume that shedding into the blood is proportional to the death rate of a tumor region [32], i.e., the fraction of a tumor clone in the ctDNA population at time *t* can be computed as a weighted average over the frequency of the clone in each region, $\frac{\sum_{j}d_{j}N_{ij}\left(t\right)}{\sum_{i}\sum_{j}d_{j}N_{ij}\left(t\right)}$.

While this represents the clone’s fraction of the tumor population, to test the effect of clone fraction distortion on mutation detection, we also estimated clinically realistic VAFs in the blood, which also contains DNA fragments from healthy tissue. To do this, we computed the frequencies of each driver mutation belonging to each clone and then estimated the fraction of the total number of fragments that originate from the tumor (the tumor fraction). At the point of diagnosis, Phallen et al. found that the mean tumor fraction in the bloodstream for stage I and II breast, lung, ovarian, and colorectal tumors was 1% [41]. We calibrated the simulated tumor fraction by assuming this is the fraction for proliferative tumors at the point of detection, assumed to occur at 3 billion cells, with an initial death rate of $d_{1}=0.1$.

To estimate a shedding probability, we adapted a formula from Avanzini et al. [32]. Assuming an exponentially growing tumor with a constant growth rate, the formula computes the number of fragments shed into the bloodstream as a Poisson-distributed random variable, with mean $C=\frac{Ndq}{\epsilon \;+\;r}$, where *N*, *d*, *q*, ϵ, and *r* are the number of cells, death rate, shedding rate, decay rate, and growth rate, respectively. We estimated C using the Phallen data set, which found the median DNA concentration in plasma to be 29 ng/mL. Repeating a calculation from their paper, a haploid genome weighs roughly 0.0033 ng, suggesting that there are 8788 haploid genome equivalents (HGEs) in 1 mL of plasma. With 5 L total blood volume in the human body and 55% plasma, we can therefore estimate *C* to be 5000×0.55×8788×0.01= 241,670. While the formula depends on *r* (the tumor birth rate can in fact slightly alter the total ctDNA molecules present in a blood draw), the tumor population changes in the order of days, while DNA decays in the blood with a half-life of about 30 min [10]. This implies ϵ=48ln2≈33.3, while r<1. In a spatial setting, the effective growth rate is even lower because cells do not divide when surrounded, so we assumed r≈0. Setting $C=\frac{3\;\times \;10^{9}\;\times \;0.1\;\times \;q}{\epsilon }$, we estimate q≈0.026. While this shedding probability is likely to vary between tumors based on location in the body, type, and exposure to blood vessels, we did not consider these in order to focus on how stochastic variation in where mutations appear and spread causes clone death rates and bias in VAFs to vary among otherwise identically growing tumors. The mean number of tumor fragments at other time points is then computed as $C_{t}=\frac{Nq\bar{d}}{\epsilon }$, where $\bar{d}$ is the mean death rate of the whole tumor. For a mutation *m* with tissue frequency $f_{m}$ and overall death rate $\bar{d_{m}}$, we wrote the total number of fragments with that mutation as $C_{m}$∼$\mathrm{Pois}(\frac{f_{m}N\bar{d_{m}}q}{\epsilon })$. For a 15 mL blood draw (0.3% of the total supply), we scaled the mean number of fragments by 0.003. Let $C_{tot_{0}}$ be the total fragments in a 15 mL blood draw at the point of detection. Then $C_{tot_{0}}$∼Pois(5000×0.55×8800×0.003). We assumed the mean number of nontumor fragments remains constant at $C_{h}=0.99*C_{tot_{0}}$. If we assume all cells are diploid, each mutation appears on a single chromosome copy and we ignore the possibility of recurrent mutation or subsequent allelic gain or loss, we can write the expression for the spatially biased VAF of a specific mutation in the blood as $\frac{\mathrm{Pois}(\frac{1}{2}C_{m})}{\mathrm{Pois}(C_{t}\;+\;C_{h})}$. To analyze the effect of spatially correlated death rates on the detection of tumor mutations, we computed both spatially biased and unbiased VAFs by using the mean death rate of the specific mutation ($\bar{d_{m}}$) for the former, and the mean death rate of the entire tumor (replace $\bar{d_{m}}$ with $\bar{d}$ in the expression for $C_{m}$) for the latter.

**Inverse Simpson diversity as a measure of intratumor heterogeneity (ITH).** Since an important goal of this work is understanding how ctDNA data collected from the blood may distort estimates of clonal heterogeneity present in the main solid tumor, we used the inverse Simpson diversity index to quantify and compare heterogeneity estimates from blood and tissue sequences. The inverse Simpson diversity index is a classic diversity measure employed in many previous studies of population diversity that takes into account the number of lineages present, as well as the relative abundance of each [38,42]. For a set of clone fractions $f_{1},\cdots ,f_{N}$, with $\sum_{1}^{N}f_{i}=1$, it is defined as $D=\frac{1}{\sum_{1}^{N}f_{i}^{2}}$.

## 3. Results

### 3.1. Spatial Differences in Apoptosis and Shedding Can Bias Clone Fractions in ctDNA

**Figure 2:**
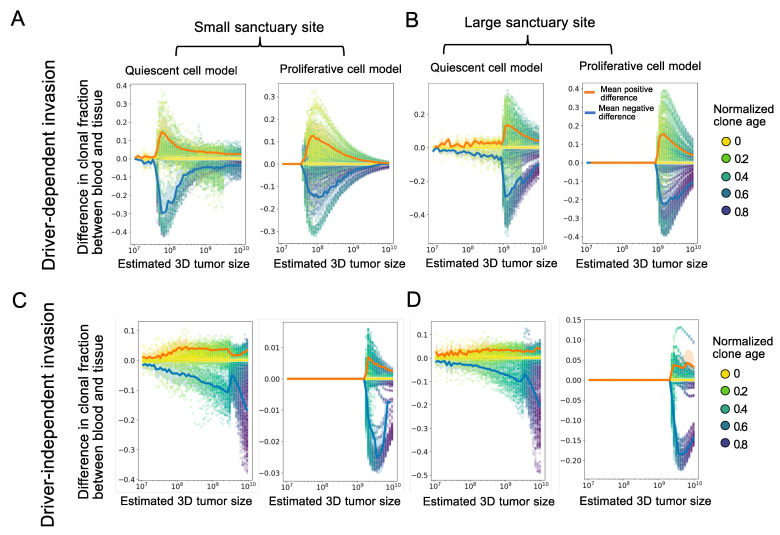
Clone fraction differences between blood and tissue: (**A**–**D**) Each plot shows the results of 50 simulation runs, where each point represents the difference between clonal frequencies estimated from the blood versus those present in the tumor, for a single clone, with the color showing the age of the clone relative to the total simulation time. Tumors were grown from a single cell until reaching a 2D cross-section of a 10-billion-cell tumor. For all simulations, $\mu =0.001,\;s=0.1,\;d_{1}=0.1,\;b=0.7$. For driver-dependent invasion, $d_{2}=0.9$. For driver-independent invasion, $d_{2}=0.69$. The orange and blue lines show the average positive and negative clone fraction difference, respectively. Only clones comprising at least 10% of the tumor were included in the average. Shading is ±1 s.d. We show the same plots over normalized time in Supplementary Figure S2.

To study how the spatial structure of a solid tumor, through spatial heterogeneity in apoptosis, can bias the observed ctDNA in blood draws, we first analyzed the difference in the clonal fractions between blood and tumor tissue. In Figure 2, we compared the results for proliferative versus quiescent cell models, small versus large sanctuary sites, and driver-dependent versus driver-independent invasion. Across all modeling scenarios, Figure 2 shows that new clones on the expanding front tend to be over-emphasized in the ctDNA, while older clones, trapped in the tumor sanctuary, tend to be under-represented. The magnitude of the differences in clonal fraction and their likelihood to impact clinical detectability depend on the accumulated clonal diversity on the edge of the tumor, mediated by the edge environmental effects.

In the driver-dependent case (Figure 2A,B), the few driver clones able to invade the new environment experience a higher death rate during expansion on the edge and end up over-represented in the blood, making the absolute difference between the blood and tissue clone fractions substantial. The maximum difference between the two occurs in the limiting case of a single clone, originating on the expanding front and growing without competition in the new edge environment. For proliferative tumors, we can write an upper bound for this clone fraction difference. If we assume the tumor initiates with death rate $d_{1}$ and grows to a constant size *S*, after which a single invasive subclone grows to size *x*, experiencing death rate $d_{2}$, the difference in the expected clone fraction can be written as

$$f=\frac{d_{2}x}{d_{2}x+d_{1}S}-\frac{x}{x+S}.$$

It is easy to show that the maximum value of *f* is $\frac{\sqrt{d_{2}}\;-\;\sqrt{d_{1}}}{\sqrt{d_{2}}\;+\;\sqrt{d_{1}}}$, which occurs when $x=S\sqrt{\frac{d_{1}}{d_{2}}}$. We plot the maximum possible clone fraction difference for all $d_{1}$ and $d_{2}$ in Supplementary Figure S1 and show that despite the apparently high choice of $d_{2}$ in some of our simulations, large differences in the estimated clonal frequencies can occur with very small absolute death rates. In line with the prediction that the peak clone fraction difference does not depend on region size, the simulations also showed that for driver-dependent invasion, the size of the tumor sanctuary does not greatly impact the distribution of clonal fraction differences (Figure 2A,B).

The sanctuary size does affect the results for proliferative driver-independent tumors, which show very little difference between the ctDNA and main tissue when the sanctuary site is small (Figure 2C). This is because early clones from the small sanctuary region can invade the edge environment before the appearance and spread of later clones and are therefore represented throughout all tumor regions that differentially shed into the blood. This effect is still present with a larger sanctuary site, since the observed minimum clonal fraction difference is still much smaller than the corresponding one in the driver-dependent case (compare Figure 2B,D).

For quiescent tumors, ctDNA can only come from the shedding of cells on the expanding front, which is determined by the total size of the tumor prior to detection, and the sanctuary size again has little effect on the observed differences (Figure 2B,D). Despite this, the magnitude of the differences in death rates are comparable to those of proliferative tumors. However, we notice that quiescent tumors distort clone fractions across all population sizes and time points due to the additional spatial bias in death rate. One thing to note is that while we assumed that differences in shedding are caused by spatial heterogeneity in death rates, we expect results to be similar in any extension of the model in which clones are weighted differently in the ctDNA than the tissue, for example, with differential access to the bloodstream based on proximity to blood vessels or via a model of active secretion. Additionally, we found that the version of the model where driver mutations reduce death rate, akin to apoptosis resistance, results in similar clone fraction distortions (Supplementary Figure S3).

### 3.2. Differential Shedding Leads to Overestimation of True Intratumor Heterogeneity

**Figure 3:**
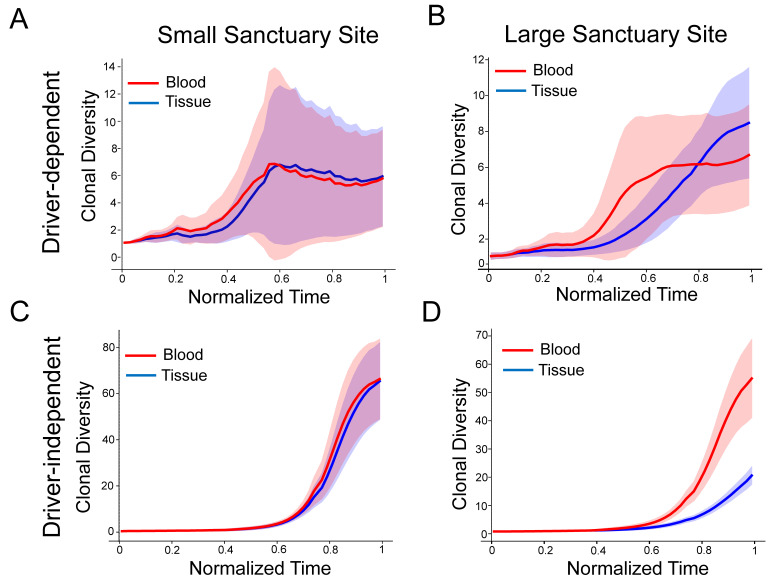
Discrepancies between blood and tissue clonal diversity: (**A**–**D**) The subplots show the inverse Simpson diversity index of the clonal frequencies in the blood and tissue for each clone in 50 simulated tumors. Timepoints are normalized by run and then binned and downsampled. Tumors were grown from a single cell until reaching a 2D cross-section of a 10-billion-cell tumor. For all simulations, $\mu =0.001,\;s=0.1,\;d_{1}=0.1,\;b=0.7$. For driver-dependent regrowth, $d_{2}=0.9$. For driver-independent regrowth, $d_{2}=0.69$. Shading represents ±1 s.d. The figure shows results for proliferative tumors only. For all scenarios, see Supplementary Figure S4.

In Figure 3, we used the inverse Simpson diversity index across normalized time points as a proxy for ITH in the ctDNA and in the tissue over the course of tumor progression. We found that driver-independent tumors with a large sanctuary site consistently show a large difference between blood and tissue ITH (Figure 3D), while tumors with a small sanctuary site do not show any difference. This is a consequence of the clone fraction differences observed in Figure 2, which, for proliferative tumors, vanish once the sanctuary site is too small. Also consistent with Figure 2, quiescent driver-independent tumors show elevated ITH for both sanctuary sizes (Supplementary Figure S4). As expected, driver-dependent tumor growth is driven by very few clones following detection, which results in much lower overall clonal diversity (Figure 3A,B and Figure S4).

### 3.3. The Effect of Sequencing Detection Limits and Sanctuary Site Size on Observed VAFs in the Blood

**Figure 4:**
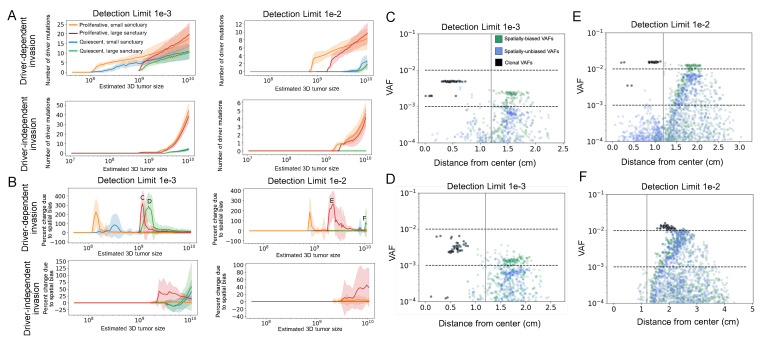
Influence of spatial bias on limits of detection. (**A**) Plots of the number of detectable driver mutations starting from the point of relapse for minimum detection frequencies of 1 × 10^−3^ and 1 × 10^−2^ for proliferative and quiescent tumors relapsing at ∼10^8^ and ∼10^9^ cells. Mutations were detectable if the estimated VAF exceeded the detection limit. VAFs were estimated based on a tumor fraction of 1% for a 3-billion-cell tumor with death rate of 0.1 (see Methods). (**B**) Percent change in number of detectable drivers when the VAFs in (**A**) are compared to VAFs computed assuming the tumor sheds all clones at the same rate for the same detection limits see Methods). (**C**–**F**) Scatter plots of mean spatially biased VAFs (green) and unbiased VAFs (blue) at the size where the average spatial bias over all replicates is maximal (marked with the corresponding letter in (**B**)) Each plot shows all mutations for 50 replicates of the corresponding simulation scenario. The x-axis is the mean distance of the mutation from the tumor’s center. Black points are clonal mutations, which show perfect overlap between the blood and tissue. The vertical line marks the end of the sanctuary region.

We next analyzed how biased clonal fractions in the blood translate into biased observed VAFs under various sequencing detection limits. In Figure 4, we considered sequence detection limits of $10^{-3}$ and $10^{-2}$, which are often utilized for panel-based assays optimized for MRD detection [43]. As expected, a higher sequence detection limit of $10^{-2}$ diminishes the number of detected drivers (VAF exceeds the detection limit) and increases the tumor size at which the first mutations are detected, compared to a limit of $10^{-3}$ (Figure 4A). This effect is more pronounced in quiescent tumors than in proliferative ones. While driver-independent tumors produce many more mutations, responsible for the higher ITH shown in (Figure 3), they are nonetheless very low frequency and so the number of mutations above a $10^{-2}$ threshold is comparable to that of driver-independent tumors. Most mutations evade detection entirely, as the overall percentage of driver mutations detected at any point is below 10% for all scenarios (Supplementary Figure S5C,D).

In Figure 4B, we compared the percent change in number of detectable drivers when the simulated VAFs are compared to VAFs from a spatially uniform null model, computed assuming the tumor sheds all clones at the same rate. We show that spatial tumor heterogeneity can greatly affect the number of detectable driver mutations in the blood, and sequencing detection limits can further alter the extent of this bias, with the timing and magnitude of difference spikes further dependent on the detection limit of the sequencing technology.

Because clonal VAFs cannot change due to shedding differences, the effect depends entirely on the detection limit relative to subclonal VAFs. We see that spatial bias in proliferative driver-dependent tumors increases when the detection limit is raised, but quiescent spatial bias either decreases in magnitude and appears at a larger tumor size or disappears all together. We show the percent spatial bias over normalized time in Supplementary Figure S5B.

In Figure 4C–F, we show the dependency of spatial bias on detection limit by plotting the frequency versus the mean tumor radius of every mutation present in 50 simulation runs at the point of maximal spatial bias (the labeled peaks in Figure 4B). Plots corresponding to the peaks of the other scenarios are shown in Supplementary Figure S6. We can observe a cluster of clonal mutations in the core of the tumor (colored black), which are equally represented in the blood and tissue. Due to boundary-driven growth, subclonal mutations accumulate more on the edge of the tumor and tend to remain there across generations, increasing the frequency of mutations further from the core. Because the mutations also shed at higher rates, filtering for larger mutations can increase bias but will decrease it once the majority of detectable VAFs are clonal (Figure 4F). Of clinical relevance is the case where subclonal variants are exaggerated to near-clonal frequencies, which occurs in the driver-dependent case (Figure 4C–F). This showcases the benefits and risks of distorted ctDNA: while exaggerated subclonal mutations would provide more biomarkers to aid in detecting recurrence, they would make poor targets for treatment.

## 4. Discussion

As cancers grow, they slough off cells and DNA from apoptotic or necrotic cancer cells, which enter the bloodstream. Through the use of technologies such as next-generation sequencing, these fragments of DNA can reveal a wealth of information about cancer, without the need for invasive surgical biopsies. Here, we explored how boundary-driven tumor growth and spatial heterogeneity in cellular death rates impact both the clonal evolution of the tumor and its representation in ctDNA. We found that the appearance of genetic distortions between blood and tissue ultimately depends on whether the tumor’s genetic heterogeneity varies with respect to rates of apoptosis and ctDNA shedding, which themselves can vary between tumors or over time for a single tumor. When there is a strong correlation, such as when a change in cellular death rate occurs in direction of tumor growth, ctDNA can drastically bias which clones are observed and can lead to biased estimates of intratumor heterogeneity (ITH).

In the driver-dependent case and, to a lesser extent, the driver-independent case explored here, this bias can be beneficial by increasing the visibility of and sensitivity for the particular mutations responsible for tumor progression. Spatial differences in cell death rates could also lead to subclonal mutations appearing at clonal frequencies in ctDNA, thus increasing the likelihood that they are mistaken for clonal mutations and chosen as therapeutic targets (Figure 4). Our results agree with findings that quiescent tumors may be difficult to detect in the bloodstream (Figure 4A) and further suggest that any detectable ctDNA is likely to dramatically under-represent some tumor regions with reduced shedding (Figure 2). One possibility is that a lesion with a quiescent interior could be nearly undetectable and suddenly begin to shed appreciably due to a clonal expansion. Because of the extremely biased location of shedding in quiescent tumors, the overall size should not be assumed to correlate well with ctDNA yield. The potential for the exaggerated observed heterogeneity in the blood relative to the tissue for tumors experiencing high apoptosis on the expanding front suggests that low-frequency clones, with a high probability of being undetected in a tissue sample, could be better captured in the blood and provide an early indicator of heterogeneous growth. At the same time, when clinical studies find greater heterogeneity in blood than in tissue samples, this is usually mainly attributed to missed heterogeneity in the tissue sample. However, localized high death rates could generate more mutations and at the same time enrich these in ctDNA through increased shedding. This is both a potential confounding factor for assessing tumor mutational burden from ctDNA and simultaneously supports the potential of blood-based diagnostics to be a more sensitive indicator of changing levels of heterogeneity than tissue biopsies. Recent work has found that in contrast to a high tissue mutational burden, which may indicate high neoantigen load and better overall survival, high blood mutational burden may better reflect overall ITH and therefore indicate poor overall survival [44]. High heterogeneity correlated to high-shedding regions could contribute to this discordance.

This general principle that genetic distortion between blood and tissue is a function of clonal dynamics is not limited to spatial heterogeneity in intrinsic death rates: it could also arise as the result of differential access to blood vessels or nutrients. Further specific scenarios can be theoretically and clinically explored, such as local metastasis of a primary breast tumor to the lymph nodes or the microinvasion of a colorectal tumor into the subserosal tissue, particularly during neoadjuvant treatment when the tumor faces novel selective pressure. In both of these cases, there is recent evidence that ctDNA shedding can vary as a function of spatial location. Clonal replacement during treatment for early-stage breast tumors is also well documented, and a small study of early-stage breast cancer patients discovered mutations private to clones that invaded the lymph nodes. In one patient, as an example of subclone over-representation, these mutations comprised the majority of detected ctDNA [45,46,47].

While our simulations considered only a single form of spatial growth and dis not incorporate a fully realistic downstream analysis of ctDNA, here, we nonetheless found that even a simple model of spatially heterogeneous tumor growth and shedding can showcase how blood sample data may not represent the tissue accurately, depending on the evolutionary processes shaping the tumor around the time of a blood draw. Further biases as a result of low tumor fraction in cfDNA, copy number variation, germline mutations, hematopoetic mutations, and heterogeneity absent from small tissue samples introduce significant additional complexity that we ignored here [48,49]. Future directions include incorporating a spatial model of blood vessel distribution that impacts drug delivery, oxygenation, and the resulting apoptosis and shedding rates. Rather than modeling changes to overall clone frequencies under an infinite sites assumption, incorporating a specific resistance model would further allow predictions of the detectability of specific drivers. If we considered resistance to a specific druggable target rather than generic driver mutations, the mutation rate would likely be much lower as it would now be site-specific, and back mutations would be possible. This would likely reduce the number of distinct resistance mutations detected and increase the waiting time to resistance. This issue could be further explored through a modified version of our driver-dependent invasion scenario. Here, we assumed that changes to birth and death rates occur incrementally through a series of point mutations, while specific models of chemotherapy resistance or immune escape may have a different effect on growth rates and the resulting shedding. Because the expanding clones in our model continue to experience high apoptosis, our results would best apply when apoptosis reduction is absent or only partial in the resistant population, such as in apoptosis-induced compensatory proliferation (AICP) [50].

A further area of study is using model insights to correct for the observed bias between ctDNA and tissue genetics. The work here revealed some of the circumstances in which we would expect such a bias to manifest and the mechanisms through which it would occur, but systematically inverting that bias to reconstruct with maximum fidelity the clonal composition of the tumor from the blood data will require further work. For example, some important applications of tumor genome samples to clonal lineage tracing (“tumor phylogenetics”) depend on accurate quantification of allele frequencies, and extending such methods to use blood data productively will require ways to not only identify but also correct for these biases. It will be important to characterize the circumstances under which this problem is invertible and what additional data might be needed. Development of models such as ours to provide accurate quantitation of tumor state from ctDNA can in turn enable new clinical applications, such as quantifying changes in tumor size or clonal composition, that are indicative of response to treatment or disease progression.

## 5. Conclusions

ctDNA can be used to reveal information about the likely presence and burden of cancer within the body. To make full use of this new technology, further work is needed to understand all of the ways that ctDNA can provide a distorted mirror of the genetic composition of the main tissue, how the evolution of the main tumor shapes these genetic biases, and how to correct for them.

## Figures and Tables

**Table 1:** Main parameters used in the model.

*N*	Final tumor size
*R*	Core / sanctuary site radius
*b*	Initial cell birth rate
$d_{1}$	Cell death rate in the tumor core
$d_{2}$	Cell death rate in the tumor edge
*s*	Driver mutation fitness advantage
μ	Poisson-distributed driver mutation rate

## Data Availability

The code and raw data used to generate all results and figures for this paper can be found at https://github.com/trachman1/lattice-tumor-ctdna (accessed on 17 January 2024).

## Supplementary Materials

The following supporting information can be downloaded at: https://www.mdpi.com/article/10.3390/cancers16050844/s1.
