# Supplementary material for: Modeling the Effect of Spatial Structure on Solid Tumor Evolution and Circulating Tumor DNA Composition
Source: Cancers (Basel). 2024 Feb 20;16(5):844. doi: 10.3390/cancers16050844 (PMC10930890; doi:10.3390/cancers16050844)
Supplement: Supplementary file 1 [file cancers-16-00844-s001.zip › cancers-2855884-supplementary.pdf]

Supplementary Material: Modeling the effect of spatial structure on solid tumor evolution and ctDNA composition

Thomas Rachman[1, 2], David Bartlett[3], William Laframboise[3], Patrick Wagner[3], Russell Schwartz[1], and Oana Carja *[1]

[1]Computational Biology Department, School of Computer Science, Carnegie Mellon University, Pittsburgh, PA, USA

[2]Joint Carnegie Mellon University-University of Pittsburgh Ph.D. Program in Computational Biology

[3]Allegheny Cancer Institute, Allegheny Health Network, Pittsburgh PA

*To whom correspondence should be addressed. Email: oana@cmu.edu; oana.carja@gmail.com

Supplementary Figures

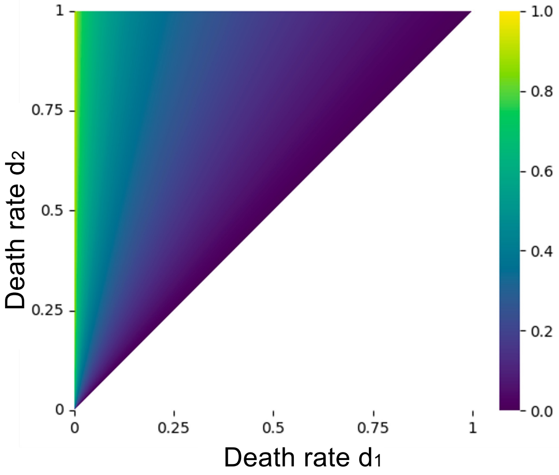

Figure S1: A heatmap showing the maximum clone fraction difference possible for proliferative tumors with respect to all values of d_1 and d_2.

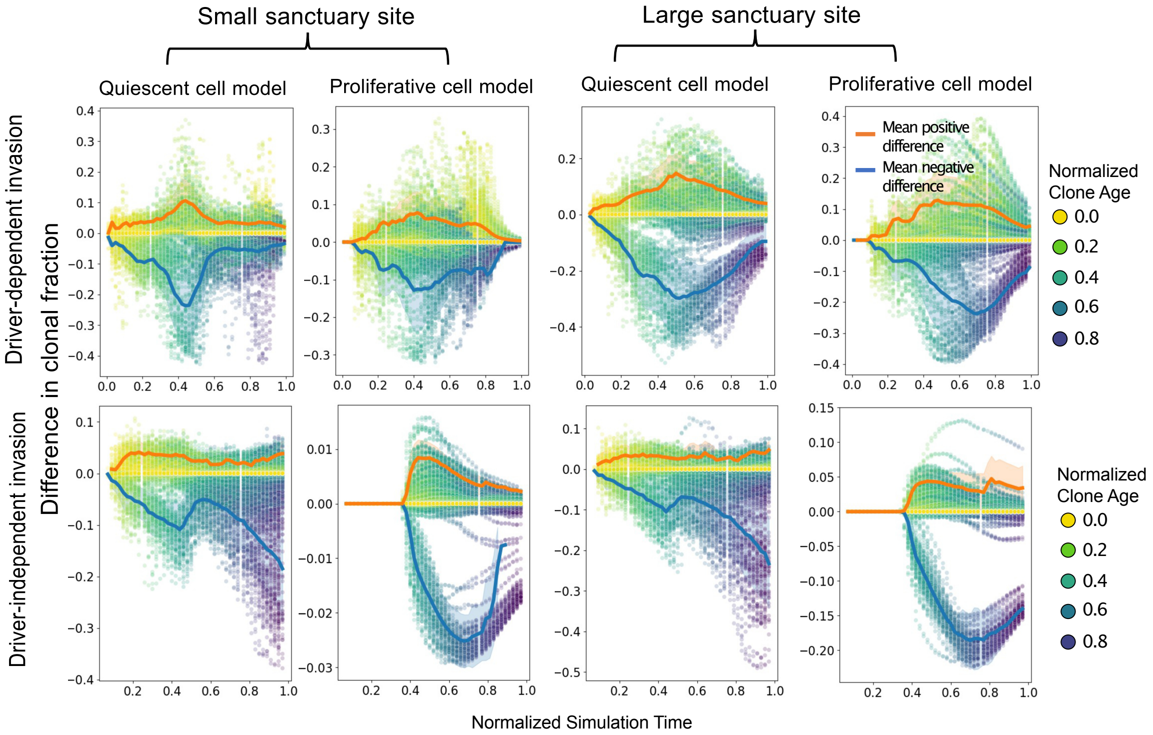

Figure S2: **Clone fraction differences between blood and tissue over normalized time: (A-D)** Each plot shows the results of 50 simulation runs, where each point represents the difference between clonal frequencies estimated from the blood versus those present in the tumor, for a single clone, with the color showing the age of the clone relative to the total simulation time. Tumors were grown from a single cell until reaching a 2D cross-section of a 10 billion cell tumor. Because mutation accumulation is random, we used down-sampled, normalized time points to plot each simulation run within a similar time frame. For all simulations, $\mu = 0.001, s = 0.1, d_1 = 0.1, b = 0.7$. For driver-dependent relapse, $d_2 = 0.9$. For driver independent invasion, $d_2 = 0.69$. The orange and blue lines show the average positive and negative clone fraction difference, respectively. Only clones comprising at least 10% of the tumor were included in the average. Shading is ± 1 s.d.

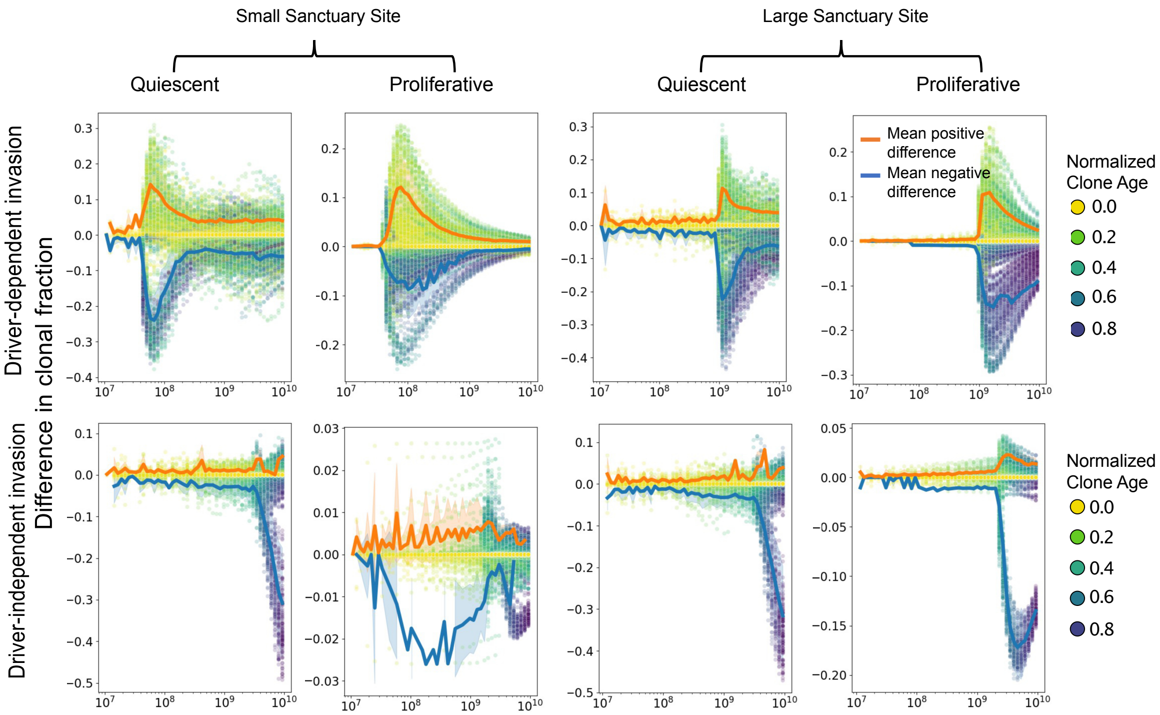

Figure S3: **Clone fraction differences between blood and tissue with selection acting on death: (A-D)** Each plot shows the results of 50 simulation runs, where each point represents the difference between clonal frequencies estimated from the blood versus those present in the tumor for a single clone, with the color showing the age of the clone relative to the total simulation time. Tumors were grown from a single cell until reaching a 2D cross-section of a 10 billion cell tumor. For all simulations, $\mu = 0.001, s = 0.1, d_1 = 0.1, b = 0.7$. For driver-dependent relapse, $d_2 = 0.9$. For driver independent invasion, $d_2 = 0.69$. The orange and blue lines show the average positive and negative clone fraction difference, respectively. Only clones comprising at least 10% of the tumor were included in the average. Shading is ± 1 s.d.

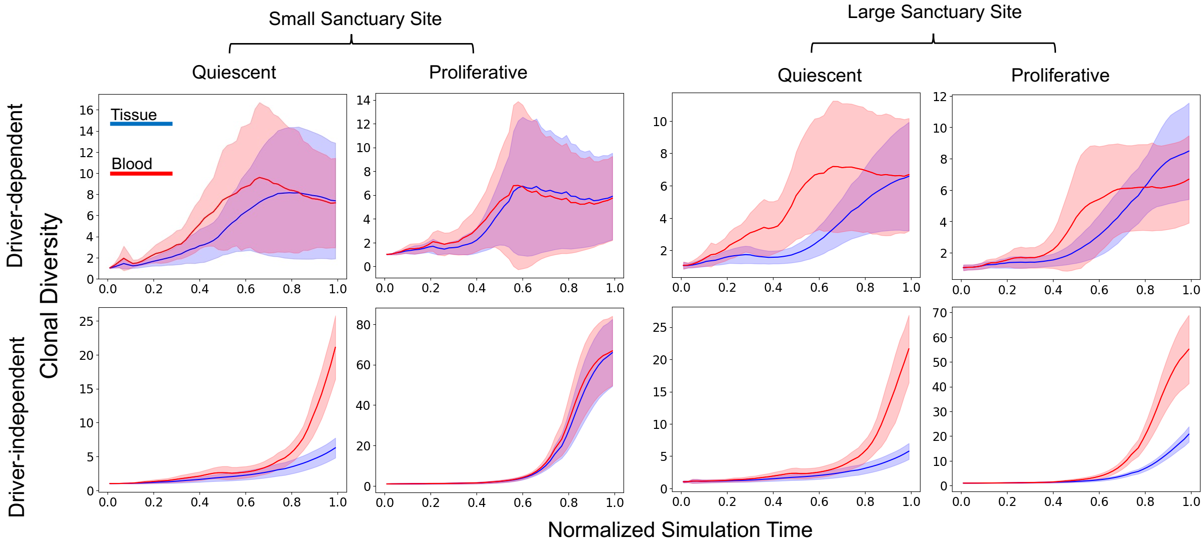

Figure S4: **Discrepancies between blood and tissue clonal diversity:** Inverse Simpson index of clone frequencies in blood and tissue for each clone in 50 simulated tumors at simulation timepoints normalized by run and then binned and down-sampled. Tumors were grown from a single cell until reaching a 2D cross-section of a 10 billion cell tumor. For all simulations, $\mu = 0.001, s = 0.1, d_1 = 0.1, b = 0.7$. For driver-dependent regrowth, $d_2 = 0.9$. For driver independent regrowth, $d_2 = 0.69$. Shading represents ± 1 s.d.

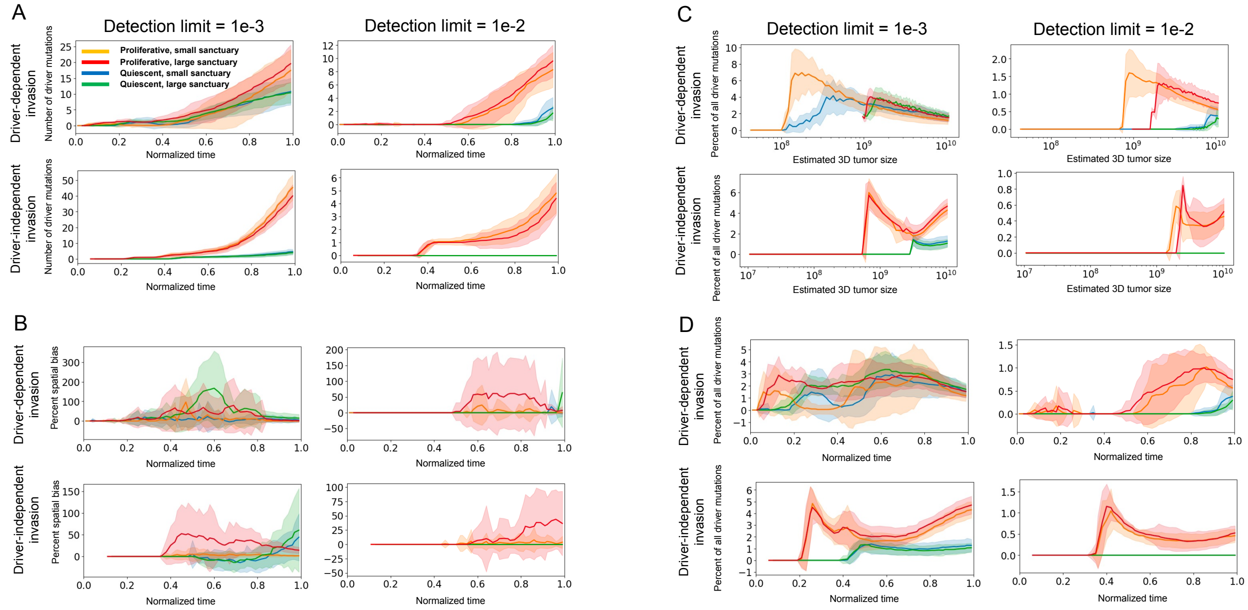

Figure S5: **Number, percent spatial bias, and overall percentage of detectable drivers: (A)** Plots of the number of detectable driver mutations starting from the point of relapse for minimum detection frequencies of 1e-3 and 1e-2, for proliferative and quiescent tumors, relapsing at $\sim 10^8$ and $\sim 10^9$ cells, over normalized timepoints. Mutations were detectable if the estimated VAF exceeded the detection limit. VAFs were estimated based on a tumor fraction of 1% for a 3 billion-cell tumor with death rate of 0.1 (see Methods). **(B)** Percent change in number of detectable drivers when the VAFs in (A) are compared to VAFs computed assuming the tumor sheds all clones at the same rate for the same detection limits, referred to as percent spatial bias (see Methods). **(C)** Overall percentage of detected driver mutations relative to population size. **(D)** Overall percentage of detected driver mutations relative to normalized timepoints.

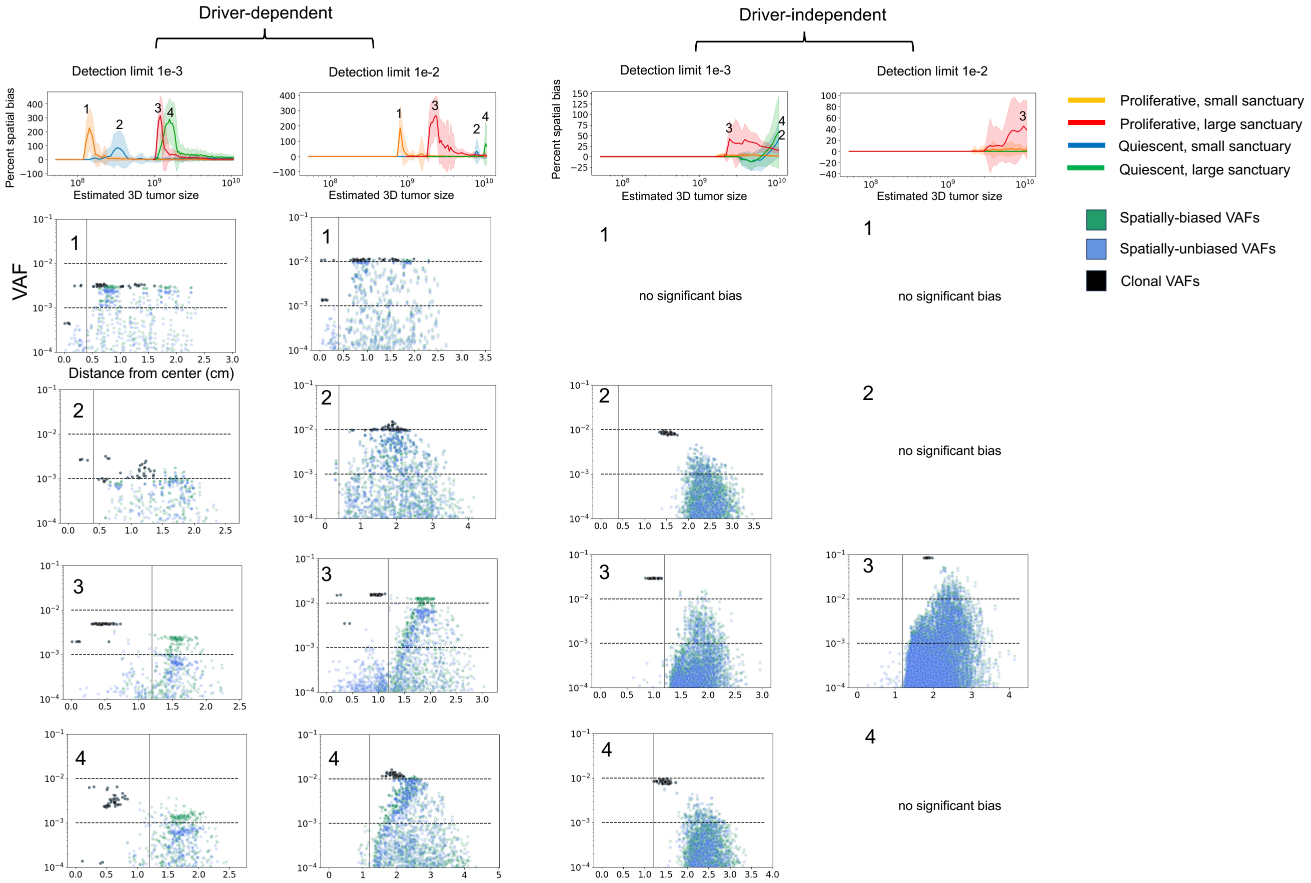

Figure S6: **Spatial distribution of VAFs at points of maximal spatial bias for all scenarios:** The top row of line plots are repeated from **Figure 4B**, showing the percent change in detected driver mutations for detection limits $1e-3$ and $1e-2$, under driver-dependent and independent invasion. Each scatterplot shows the distribution of VAFs corresponding to distance from the tumor center.
